# ESCAPE: database for integrating high-content published data collected from human and mouse embryonic stem cells

**DOI:** 10.1093/database/bat045

**Published:** 2013-06-21

**Authors:** Huilei Xu, Caroline Baroukh, Ruth Dannenfelser, Edward Y. Chen, Christopher M. Tan, Yan Kou, Yujin E. Kim, Ihor R. Lemischka, Avi Ma'ayan

**Affiliations:** ^1^Department of Pharmacology and Systems Therapeutics, Icahn School of Medicine at Mount Sinai, One Gustave L. Levy Place, Box 1215, New York, NY 10029, USA, ^2^Department of Developmental and Regenerative Biology, Icahn School of Medicine at Mount Sinai, One Gustave L. Levy Place, Box 1215, New York, NY 10029, USA, ^3^Systems Biology Center New York (SBCNY), ^4^Black Family Stem Cell Institute, Icahn School of Medicine at Mount Sinai, One Gustave L. Levy Place, Box 1215, New York, NY 10029, USA

## Abstract

High content studies that profile mouse and human embryonic stem cells (m/hESCs) using various genome-wide technologies such as transcriptomics and proteomics are constantly being published. However, efforts to integrate such data to obtain a global view of the molecular circuitry in m/hESCs are lagging behind. Here, we present an m/hESC-centered database called Embryonic Stem Cell Atlas from Pluripotency Evidence integrating data from many recent diverse high-throughput studies including chromatin immunoprecipitation followed by deep sequencing, genome-wide inhibitory RNA screens, gene expression microarrays or RNA-seq after knockdown (KD) or overexpression of critical factors, immunoprecipitation followed by mass spectrometry proteomics and phosphoproteomics. The database provides web-based interactive search and visualization tools that can be used to build subnetworks and to identify known and novel regulatory interactions across various regulatory layers. The web-interface also includes tools to predict the effects of combinatorial KDs by additive effects controlled by sliders, or through simulation software implemented in MATLAB. Overall, the Embryonic Stem Cell Atlas from Pluripotency Evidence database is a comprehensive resource for the stem cell systems biology community.

**Database URL**: http://www.maayanlab.net/ESCAPE

## Introduction

Embryonic stem cells (ESCs) are pluripotent cells characterized by their capability to self-renew and differentiate into all adult cell types. Recent efforts in systematically profiling ESCs have yielded a wealth of high-throughput data. High-throughput technologies including gene expression microarrays, RNA sequencing, chromatin immunoprecipitation followed by deep sequencing (ChIP-chip/seq), genome-wide inhibitory RNA (RNAi) screens, immunoprecipitation followed by mass spectrometry (IP-MS) proteomics and phosphoproteomics, as well as other emerging technologies have been applied to profile the same cell type by many laboratories across the world in the past decade. Several databases and tools have been published to facilitate the integration of such data (1–9), and such efforts pave the way toward an *in silico* reconstruction of the gene and protein regulatory networks that regulate self-renewal and pluripotency of these important cells. For example, Plurinet (2), FunGenES (4), StemBase (5), SyStemmCell (10), iScMiD (9) and PluriNetWork (1) incorporate stem cell data from several studies and provide web-based interfaces for data query and visualization. However, in general, these databases contain information from a single regulatory layer, mostly transcriptome measurements, and thus overlook other important layers as well as cross-layer interactions. To address the need for further data integration in the field, we constructed a more inclusive database called Embryonic Stem Cell Atlas from Pluripotency Evidence (ESCAPE). This database integrates numerous additional types of data ranging from epigenetics, transcriptomics, to proteomics and phosphoproteomics. These data sets are processed into gene lists, gene–gene and protein–protein interactions, and data tables for easy download and manipulation. In addition, a rich-content web-based application has been developed to enable users to interact with the various types of data in the ESCAPE database. These tools enable users to construct subnetworks, perform enrichment analyses visualized on a canvas and predict lineage specification based on *in silico* gene KDs or overexpressions.

## Results

### A comprehensive embryonic stem cell database constructed from published high-throughput studies

**Figure 1.**
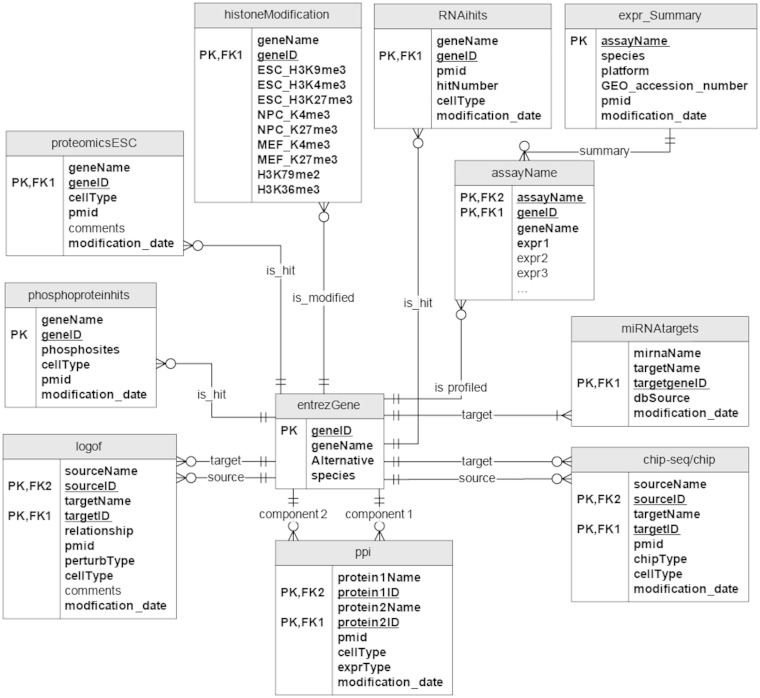
Entity relation diagram of the ESCAPE database. PK- primary key, FK- foreign key.

**Figure 2.**
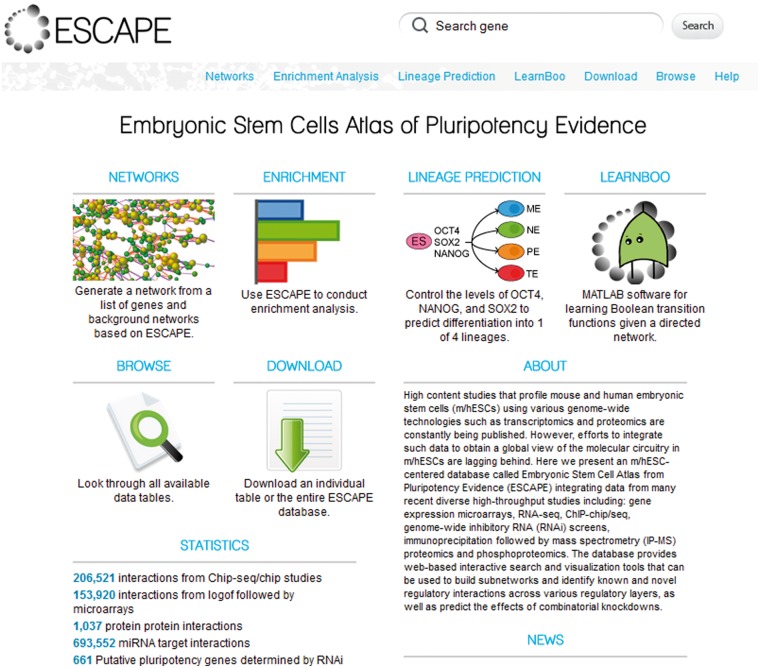
Screenshot of the main menu of ESCAPE.

Results from numerous published mouse and human embryonic stem cells (m/hESC) genome-wide profiling studies, as well as loss-of-function/gain-of-function (LOF/GOF) studies, were systematically collected and processed to construct the ESCAPE database. Most data sets are from mouse with several from human embryonic stem cells. In its current version, ESCAPE contains (i) 206 521 documented protein/DNA interactions from ChIP-chip/seq studies, connecting 61 transcription factors (TFs) to their putative target genes; (ii) 153 920 LOF/GOF interactions connecting 28 TFs from LOF KD/knockout studies followed by genome-wide expression, and 55 TFs from GOF overexpression studies followed by genome-wide expression. These interactions directly or indirectly connect a target gene to an upstream TF regulator. These interactions are directed (arrow from the factor to the target) and signed (activation/inhibition); (iii) 1037 protein–protein interactions from IP-MS interactome studies centered on 16 bait proteins, as well as from smaller-scale studies; (iv) 813 gene-products functionally identified in five large-scale RNAi screens as key regulators of mESC pluripotency; (v) 19 801 m/hESC and differentiating progeny-specific nuclear proteins from whole nuclear MS proteomic analyses; (vi) 8323 ESC and differentiating progeny-specific phosphoproteins with identified phosphosites extracted from four studies; (vii) three genome-wide microarray mRNA time courses collected during mESC differentiation from one study; (viii) one genome-wide microRNA (miR) expression data set collected from mESCs; and (xi) 18 genome-wide ChIP-chip/seq histone modification studies in ESCs and early differentiated cells. The ESCAPE database descriptive statistics are provided in Table 1. The references are also listed in Table 2. The entity relationship diagram of the database design is shown in Figure 1. Data sets to construct the ESCAPE database are freely downloadable and searchable online. The ESCAPE database is stored as a MySQL relational database. The web interface is implemented as a set of PHP scripts running under Apache as well as a set of Java Servlets running under Tomcat all interacting with the database using SQL. The network viewer used in the network generator page is Cytoscape Web (11) implemented in Flash. The canvas visualization within the enrichment analysis page is implemented with the JavaScript library D3 (12). JavaScript and AJAX are implemented throughout the site for improving user experience (UX) (13). The web interface contains several modules: (i) an interface to browse and query the data; (ii) an interface to download the data; (iii) a tool to generate subnetworks from an input list of genes using background networks generated from the database; (iv) a tool to perform enrichment analysis on user entered gene lists using background lists of genes generated from the database and visualized on a canvas, as well as enrichment analysis of user inputted lists using Enrichr, a tool to visualize enrichment results against 35 gene set libraries (14); (v) an interface to predict lineage commitment on gene KDs or overexpressions; (vi) a downloadable MATLAB software with a graphical user interface for learning Boolean functions and simulating subnetwork dynamics given a prior subnetwork topology and experimental measurements of subnetwork node expression levels across many conditions (Figure 2). Details of the modules are described in the following sections.

**Table 1.** Statistics of the current version of the ESCAPE database

Data type	Description	Interactions	Source Proteins	Studies
Protein–gene promoter binding	Protein–gene promoter binding interactions extracted from ChIP-chip/seq studies	206 521 (mESC);	61 (mESC)	38 ^a^
Protein–gene transcriptional regulation	Protein–mRNA regulatory interactions extracted from studies of LOF/GOF and GOF followed by microarray profiling	150 251 (mESC); 3669(hESC)	73 (mESC); 3 (hESC)	25(mESC); 3(hESC)
Protein–protein interactions	Protein–protein interactions from both high-throughput mass-spectrometry proteomics studies using key TF regulators as bait and manually curated from low-throughput studies	912 (mESC,HT); 118 (mESC,LT); 7 (hESC,LT)		7 (mESC,HT); 77 (mESC,LT); 4 (hESC,LT)
miRNA-predicted target interactions	miRNA-predicted target interactions in mice integrated from published databases: TarBase, TargetScan, miRander and miRBase	693 552		
Histone modifications	Histone modifications profiles of genes		107 types	6
Pluripotency genes	Potential pluripotency associated genes from multiple large-scale RNAi screen studies		640 (mESC); 173 (hESC)	9 (mESC); 1 (hESC)
ESC-specific proteins	Undifferentiated and differentiating ESC specific proteins		5547 (mESC); 6764 (hESC); 457 (diff-mESC);7033 (diff-hESC)	2 (mESC); 4 (hESC)
ESC-specific phosphoproteins-phosphosites	Undifferentiated and differentiating ESC specific phosphoproteins with specified phosphosites		3697 (hESC) 4626 (diff-hESC)	4 (hESC)
Gene expression	Time-course gene expression of ESCs under undirected differentiation			1 (3 datasets)
miRNA expression	miRNA expression in ESC/MEF/iPSC			1 (1 dataset)

^a^Including one unpublished data set for Esrrb.

**Table 2.** ESCAPE literature references

Interaction types	PMIDs	Interaction types	PMID	Interaction types	PMIDs
ChIP-chip/seq	19251738	LOF/GOF	22210892	Protein–protein interactions	19036726
	19339689		22196727		19056487
	16625203		20720539		19117947
	17442700		22327834		19158397
	18264089		22020125		19172185
	18358816	Protein–protein interactions	20362541		19349578
	19079543		20362542		19421146
	16518401		17093407		19440552
	18347094		22083510		19489080
	18692474		20946988		19536159
	18700969		1769609		19544440
	18959480		8939963		19564334
	19796622		10849651		19571885
	19030024		11791180		19625610
	18555785		11934987		19650037
	18804426		12145208		19703396
	19587682		12646244		19740739
	19884257		12774123		19798101
	19884255		14551209		19816951
	18467660		15103331		19821493
	20064375		15861132		20075857
	20075857		15863505		20110566
	20946988		16129412		20508149
	18974828		16253997		20736927
	20872845		16325584		18568018
	20139965		16382133		9748258
	21062744		16395332		21062744
	21170310		16631596		21159818
	21183938		16702210		21589869
	20123909		16702404		21884934
	21448134		16763566		22334693
	20581084		16790473	Pluripotency genes from genome-wide RNAi screen	16767105
	20144788		16801560		19345177
	21632747		16840789		19339689
	22325148		16978048		18614019
	20720539		16999741		20720539
	23239880		17030610		20953172
LOF/GOF	16518401		17324942		22143885
	16767105		17339329		22899353
	17339329		17372190		22327834
	17448993		17520687		21874018
	17515932		17543867	Proteomics	16600995
	18264089		17687327		19664995
	18757296		17892859		19151416
	19060217		17938196		21406692
	19136965		17994007		21149613
	19530134		18055446	Histone modifications	19884255
	19618472		18055449		17603471
	19884255		18223644		18692474
	20075857		18454139		18600261
	20139965		18454140		20682450
	21915945		18454141		20944595
	20875108		18462200	Phosphoproteomics	19664994
	20526341		18467660		19664995
	19796622		18585351		19151416
	20953172		18680430		21406692
	20123909		18687992	Gene expression	17394647
	21589869		18818694	miRNA expression	18692474
	21632747		18957414		
	21924763		18983969		

### Browsing and querying data sets within the ESCAPE database

The ESCAPE database provides web-based user interface to allow easy browsing and querying. From the Browse page of the web interface, users can click on one of the tables listed on the left, and then the contents of the selected table are displayed in the center of the page. The contents of the table can be sorted by clicking the name of the column. In addition, information about the methods used to generate the table and the number of entries are displayed above each table. There are two ways to search the ESCAPE database: (i) a general search for a gene using the search bar displayed on top of any web page of the ESCAPE web interface or (ii) a detailed search within a selected table. The detailed search is provided under the Browse section of the website. In the case of looking for a specific gene name using the global search, a list of all the tables where the gene appears is displayed in the search results page, and direct links to the table are provided. In the second case when searching within a specific table, more complicated queries can be created. For each column of the table, there is a possibility to choose an operator on the column, for example, equal or not equal. In addition, logical operators are provided to select specific records using the operators AND or OR. For instance, if you are searching for all the interactions where NANOG, ESRRB and SOX2 are the source genes, and the interactions are upregulation, and the experiment type is GOF, the parameters are set as follows:

1. Operator = and ‘NANOG, ESRRB, SOX2’ listed in the GeneName column.
2. Logic operator AND, and operator = and where ‘1’ is written in the Relationship column.
3. Logic operator AND, and operator = and where ‘GOF’ is written in the PerturbType column.

The query will be automatically converted to the SQL statement: ‘SELECT * FROM logof WHERE (sourceName=“srrb” OR sourceName=“Nanog” OR sourceName=“Sox2”) AND (relationship=“1”) AND (perturbType=“GOF”)’. The results will be formatted and displayed in a results page.

All the tables of the ESCAPE database can be freely downloaded from the Download page of the website. The tables are provided as either flat tab-separated text files or as mySQL files.

### Subnetwork construction with ESCAPE

**Figure 3.**
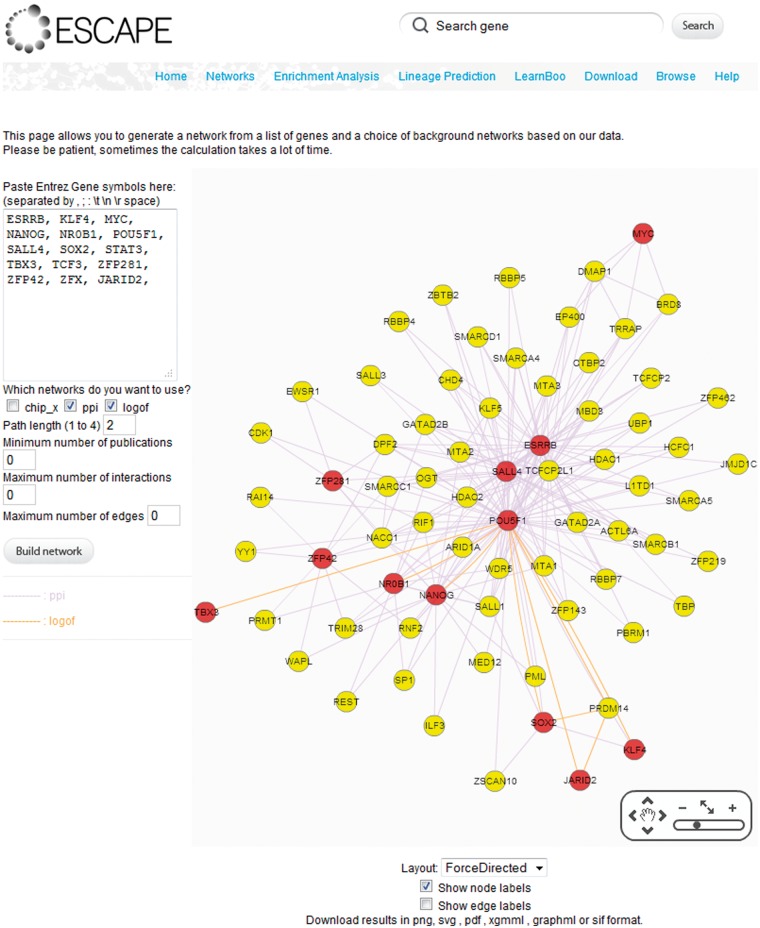
An example from the ‘NETWORKS’ tab for linking seed nodes through other nodes based on the interactions within the database.

The web interface provides a subnetwork construction functionality to facilitate connecting genes/proteins of interest using the various types of gene–gene interaction networks from the ESCAPE database and a user provided list of input gene IDs (Figure 3). The tool allows users to construct a subnetwork from a list of seed genes. The links that establish the connections within the subnetwork are determined by the background knowledge networks selected from various gene–gene interaction tables or a combination of them. Interactions in these subnetworks can be from: (i) ChIP-chip/seq, (ii) protein–protein interactions or (iii) LOF/GOF evidence. First, the user inputs a seed gene list in Entrez gene symbol format. Then, the user chooses which background networks to use to connect the seed genes. The program uses the interactions from these networks to find connections between the input seed gene list using the shortest path algorithm. The user can also select the path length between seed nodes. The default path length is set to two, or in other words one intermediate node. The program can filter interactions based on user defined parameters of minimum number of references per interaction or by maximum number of interactions per reference. Furthermore, the intermediates are ranked by significance of specificity to interact with the seed nodes as implemented by our software tool Genes2Networks (15). The resultant subnetwork is visualized using the Flash-based interactive network viewer Cytoscape Web (11) that is embedded within the web page. Based on the various interaction types, edges are colored by the various three possible types of interactions. Additionally, the output sub-networks are made available for download in PNG, SVG, PDF, XGMML, GRAPHML or SIF formats. This provides compatibility with other network visualization software such as Cytoscape (16) and yED (http://www.yworks.com/en/products_yed_about.html).

### Enrichment analysis with ESCAPE

**Figure 4.**
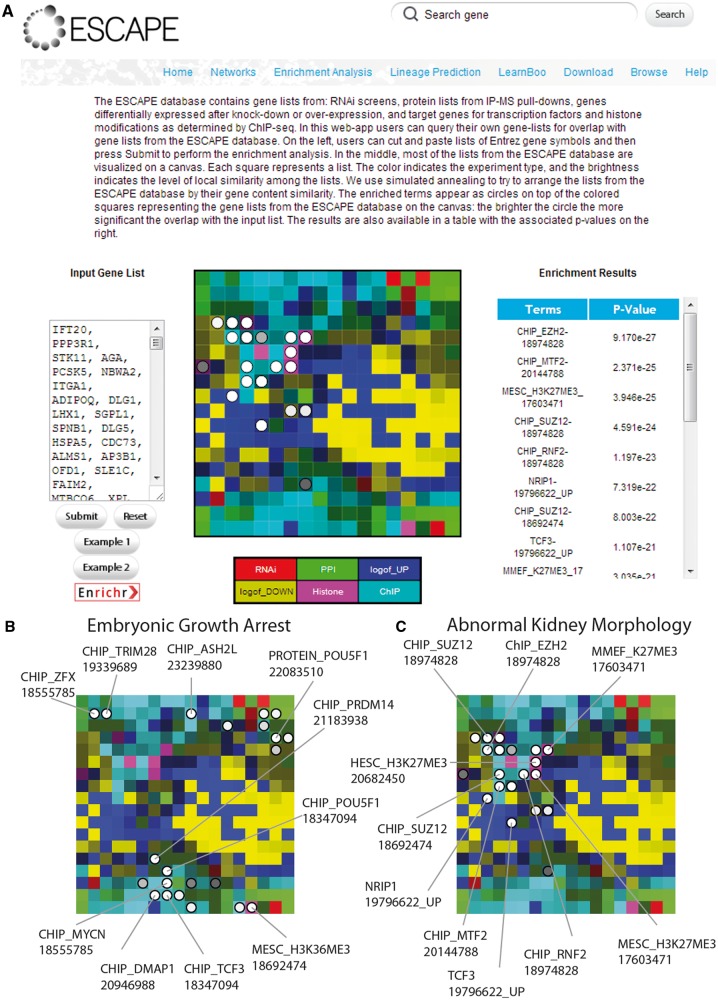
(**A**) General example from the ‘ENRICHMENT ANALYSIS’ tab for identifying overlap between gene lists from the database and other user inputted lists. (**B**) Enrichment results for a list of gene from MGI-MP with a phenotype designation ‘embryonic growth arrest’. (**C**) Enrichment results for a list of gene from MGI-MP with a phenotype designation ‘abnormal kidney morphology’. Enriched terms are highlighted in circles and some terms are annotated. Brighter circles represent more significant overlap.

Another function of the ESCAPE web interface is the ability to perform enrichment analyses (Figure 4A). The enrichment analysis tool performs gene list enrichment analysis using the various experimental modalities that produced gene lists. These include candidate genes from RNAi screens, protein lists from IP-MS pull-downs, genes differentially expressed after KD or overexpression, and target genes for TFs and histone modifications as determined by ChIP-seq/chip. In this web application portion of the site, users can query their own gene lists for overlap with gene lists from the ESCAPE database or analyze their gene list with another external gene list enrichment analysis tool called Enrichr (14). On the left, users can cut and paste lists of Entrez gene symbols and then press Submit to perform the enrichment analysis. In the middle, most of the lists from the ESCAPE database are visualized as a canvas. Each square represents a list. The color indicates the experiment type, and the brightness indicates the level of local similarity among the lists. We use simulated annealing to arrange the lists from the ESCAPE database by their gene content similarity using the Sets2Networks algorithm (17). The enriched terms appear as circles on top of the colored squares representing the gene lists from the ESCAPE database on the canvas: the brighter the circle, the more significant the overlap with the input list. The results are also available in a table with the associated p-values on the right. To compute statistical enrichment, the Fisher exact test is implemented. The resulting lists of enriched experiments only show the enriched terms determined by a cutoff threshold *P*-value of *P* < 0.05.

We created two examples to demonstrate how the enrichment analysis with the canvas visualization can be informative for obtaining new insights. We took two lists of genes that when knocked out in mice are causing the phenotypes of ‘embryonic growth arrest’ and ‘abnormal kidney physiology’ based on the MGI-MP ontology (18) terms 1730 and 2136 respectively. The enrichment results for ‘embryonic growth arrest’ show that the enriched terms are clustered in few specific areas on the grid (Figure 4B). The clustering of enriched terms is clearly not random. The input genes contain H3K36ME targets that are also Oct4 interacting proteins. Interestingly, there is also high overlap with TCFC2L1 interacting proteins as determined by proteomics and target genes of TCFC2L1 as determined by ChIP-seq. The enrichment results for ‘abnormal kidney morphology’ are all clustered in the same area, which mostly represents the PRC2 complex members, known to suppress the expression of genes required for terminal differentiation, including those critical genes for the maintenance of kidney morphology (19) (Figure 4C). Overall, such analyses can be used to link relevant phenotypes to specific regulatory mechanisms in embryonic stem cells, as well as help experimental stem cell biologists who perform high throughput experiments to place their results in context of prior studies.

### Lineage specification prediction with ESCAPE

**Figure 5.**
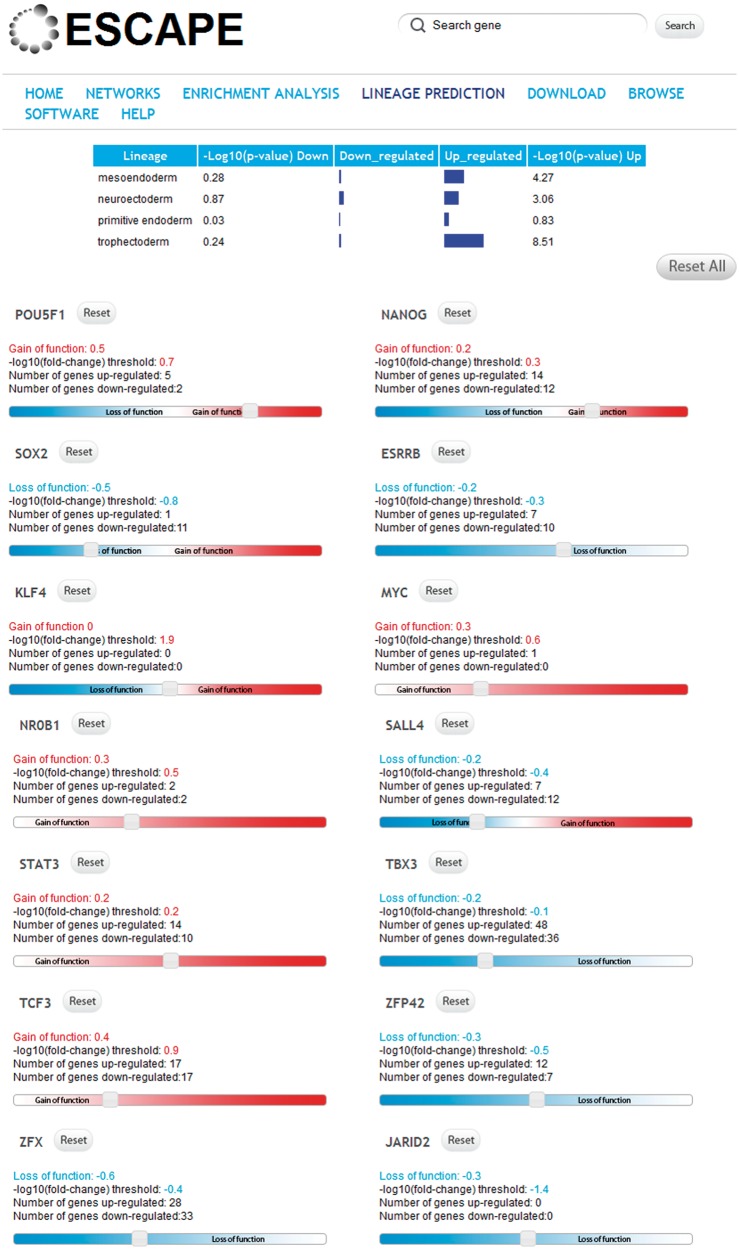
An example from the ‘LINEAGE PREDICTION’ tab showing that LOF of Oct4/Pou5f1, Nanog and Sox2 results mostly in differentiating toward the trophectoderm lineage.

The next function of the ESCAPE web interface is a tool to predict lineage-propensity differentiation outcome on single or combinatorial KD of multiple pluripotency factors (Figure 5). The tool considers the target genes of knocked-down pluripotency factors and predicts the additive expression of lineage markers based on the combinatorial additive predicted levels of these factors. Specifically, effects of gene KDs on lineage commitment are dynamically computed by enrichment analysis for targets of knocked-down factors against lists of lineage-specific marker genes using the Fisher’s exact test. Targets of KD factors were first identified from the LOF/GOF table, and lineage specific components were assembled manually from literature as follows: (i) Trophectoderm: the gene expression data set (GSE11523) reported trophectoderm-like state after depletion of Oct4/Pou5f1 in mESCs. Gene expression was profiled at six time points. Genes were sorted according to average fold change of expression on differentiation related to time point 0. The top 5% of genes with an average fold change of at least two and with a monotone increase in expression at each time point upon differentiation were considered as trophectoderm markers. (ii) Primitive endoderm: the same set of experiments and data processing as described for (i) were conduct after overexpression of *Gata6* in mESCs. (iii) Neuroectoderm: the gene expression dataset (GSE12982) isolated Sox1-GFP positive cells from mESCs where Ezh1 and Ezh2 were knocked-down. Genes were sorted according to fold change increase in expression comparing differentiated cells to mESCs. The top 10% genes with a monotonic increase and fold change of at least 1.5 were considered as neuroectoderm markers. (iv) Mesendoderm: the same set of experiments and data processing as described for (iii) were conduct after isolation of T-GFP positive cells (T stands for the gene *brachyury*). By sliding the bars on the web interface, users can choose the components and level of knockdown of 14 pluripotency factors. Corresponding positive and negative targets of each specific pluripotency factor were extracted from the LOF table within the ESCAPE database. As a result, the enrichment *P*-values reflecting the significance of differentiation potential toward each specific lineage on knockdown(s) are displayed on top. In addition, the up and down genes are provided in two text boxes below the lineage prediction display. Such lists can be further analyzed using the external enrichment analysis tool Enrichr (14) or any other tool available within ESCAPE or beyond.

### Functional associations among 15 pluripotency regulators and 15 lineage markers

**Figure 6.**
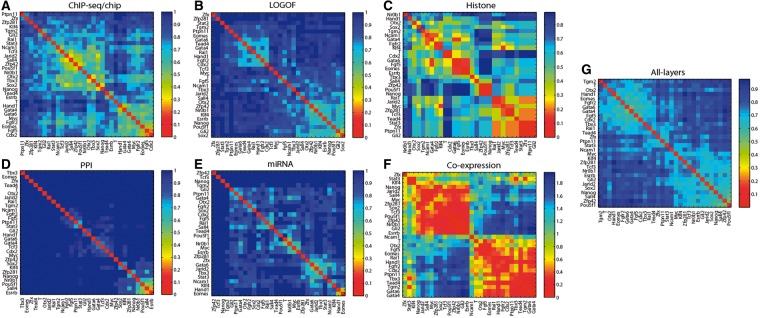
Heatmaps of 15 pluripotency components and 15 lineage marker genes. (**A–G**) Numeric values in the color bars represent similarity distance scores (1 – Similarity). Similarity is calculated using the Jaccard-coefficient for: (A) TF/target-gene promoter binding set overlap determined by ChIP-chip/seq publications; (B) Transcription-factor/mRNA regulatory interactions determined by LOF/GOF followed by expression; (C) Histone modifications determined by ChIP-chip/seq; (D) Protein–protein interactions from IP/MS studies; (E) miRNA-target gene interactions. (F) Similarity of pair-wise genes from mRNA co-expression was calculated using the Pearson’s correlation coefficient. (G) Multi-layer heatmap was constructed by normalizing the distances across all six layers.

The aggregated data in ESCAPE can be used to elucidate functional associations among pluripotency and differentiation components across various regulatory layers. Specifically, to demonstrate the usefulness of the compiled ESCAPE database to dissect the pluripotency machinery, we examined functional correlations among 15 pluripotency factors and 15 differentiation markers selected (20). Heatmaps of degree of similarity were constructed (Figure 6) where we scored pair-wise similarity distance between the components as follows: (i) Shared targets from the ChIP-chip/seq experiments; (ii) Co-expression similarities based on global mRNA measurements after pluripotency TF LOF or GOF; (iii) Histone modification target gene similarities analyzed in mESCs and differentiated cells; (iv) Protein co-occurrence measured after pull-downs of pluripotency TFs followed by MS proteomics; (v) Similarities of miR targets predicted computationally and limited to miRs preferentially expressed in mESCs; and (vi) co-expression similarities during embryoid body differentiation. Additionally, a multi-layer heatmap integrating all six layers was created. As expected, pluripotency regulators and differentiation markers generally cluster into two separate groups. A previous attempt to generate a heatmap for 13 pluripotency regulators based solely based on genomic target binding similarities resulted in slightly different clusters (21). Here, Oct4/Pou5f1 shares greatest functional similarity with Sall4 and Zfp42 (also called Rex1) (Figure 2G). This is consistent with a report that Sall4 and Oct4/Pou5f1 form a regulatory feedback loop (22). In addition, *Rex1* is a known target of Oct4/Pou5f1. However, it is interesting that *Rex1* is so closely associated with Oct4/Pou5f1 across several layers. Surprisingly, *Gli2*, a known ectoderm marker, is highly correlated with pluripotency components across numerous layers, suggesting a function in the pluripotent state for this gene. Gli2 is a downstream TF effector of Hedgehog signaling (23), and thus, potentially linking this pathway to pluripotency. Binding of Gli1 and Gli2 to the *Nanog* regulatory sequences in neural stem cells has been reported (24). Based on a recent genome-wide RNAi screen, another member of the Gli family, Gli3, was among the hits of genes involved in mESC early differentiation (25).

## Conclusions

ESCAPE is a freely available online resource that integrates current genome-wide data encompassing several regulatory layers and data types. Through the web interface, the data sets can be browsed, searched and downloaded. Additionally, a set of web-based tools were implemented to interact with the information in the database. Given a set of seed genes, users can perform network expansion, and upstream regulatory factors and downstream targets enrichment analyses, as well as perform combinatorial lineage predictions. Organizing the experimental data into a coherent and interactive framework can potentially enable better utilization of such data for systems-level analyses and construction of dynamical models. ESCAPE has been proven useful already to at least one research study (26).

## Funding

This work was supported by NIH grants R01GM098316-01A1, P50GM071558-03, R01DK088541-01A1 (A.M.) and R01GM078465-03, RC1GM091176-01 (I.R.L.). Funding for open access charge: Irma T. Hirschl Career Scientist Award (A.M.).
